# Cystic Change in the Mucous Membrane of the Stomach of a Horse

**Published:** 1881-04

**Authors:** 


### IV.—CYSTIC CHANGE IN THE MUCOUS MEMBRANE OF THE STOMACH OF A HORSE.

---

Editor Journal of Comparative Medicine:—

In October, 1878, I was called to see a sorrel mare, aged sixteen, and said to have colic. On arriving at the "*Forest House Stables*," I found my patient lying outside, where they had led her to die. After noting the symptoms and examining the case, I requested her history, which ran somewhat as follows:

She had been turned out to pasture in June, because of these spells when worked.

She showed no signs of illness when at pasture. These attacks had continued for two years, during which time she had raised a colt. The night previous she was taken from pasture and fed on new corn, with an allowance of hay. The next morning she received oats, and was then harnessed and driven to this city, a distance of nine miles. When within one-half mile of town she began to perspire profusely, and showed a disposition to lie down, but by aid of the whip was forced to her destination, where I saw her about one hour after her arrival.

Believing the case hopeless, I requested that she be killed; but before my orders could be carried out, she died.

The symptoms did not vary materially from those usually presented in gastric impaction at an advanced stage.

*Post-mortem.*—Unable to conduct this myself, it was left to the *knacker*, who related the following: On opening the abdominal cavity, the small intestines were noticed to have a slight blush, that of the duodenum being more extensive, and approaching inflammation, while the stomach at the pylorus was extremely friable. On opening this organ and removing its contents, he saw a cluster of grape-like sacs, as if strung together, and numbering some twenty or thirty independent cysts. They were removed, with a portion of the muscular walls of the stomach, and brought to me for inspection.

The mass was situated anterior to the pyloric ring in the right or lesser cul-de-sac, and hung suspended by a continuation of the mucous membrane, which was much thickened; this abnormal growth acting in time of chymification as a valve to the pyloric ring, rendering further digestion impossible.

These cysts varied in size from a hickory nut to a large hen's-egg, and when opened by the knife, there exuded a semi-gelatinous fluid resembling mucilage; its walls were about one-eighth of an inch in thickness.

P. S.—Will the Editor of the Journal be kind enongh to state whether such has been found in any other organ than the *uterus?* I called several physicians' attention to it here, but none were able to say; but pronounced it a hydatid.

Very respectfully,

Scranton, Pa. L. E. Wheat, V.S.

---

[Editorial Note.—The large number and size of the gastric tumors in this interesting case of Dr. Wheat makes the account a valuable one, and it deserves to be placed on record. In the absence of similar data from the field of veterinary medicine, one naturally turns to human diseases, which appear to furnish the required information. Polypoid excrescences, varying in size, are sometimes found in the human stomach, according to Klebs and Leube, and a very detailed account has been given by Virchow in his famous work (Die Krankhaften Geschutste, i. s., 243) of a case where they were found in the intestines.

It would appear that the gastric or intestinal follicles may undergo a mucoid change, and increase greatly in size, so that in fact they cannot remain in the gastric walls, and are expelled by muscular action. A certain amount of gastritis is usually associated with such polypi, and there is a corresponding thickening of all the coats, which in all probability is one of the earliest changes.]

